# Evaluation of potentially toxic elements and microplastics in the water treatment facility

**DOI:** 10.1007/s10661-024-12651-w

**Published:** 2024-04-25

**Authors:** Mansoor Ahmad Bhat, Agata Janaszek

**Affiliations:** 1 Government Higher Secondary School Salamabad Uri 193123, Baramulla Kashmir, India; 2 Faculty of Environmental Engineering, Geomatics and Renewable Energy, Kielce University of Technology, 25314 Kielce, Poland

**Keywords:** Microplastic, Water treatment plant, Poland, Heavy metal, Drinking water pollution, Health risk assessment

## Abstract

The potentially harmful effects of consuming potentially toxic elements (PTEs) and microplastics (MPs) regularly via drinking water are a significant cause for worry. This study investigated PTEs (Cd, Cu, Cr, Ni, Pd, Zn, Co), MPs, turbidity, pH, conductivity, and health risk assessment in the water treatment plant in Kielce, Poland. Zn had the highest concentrations throughout the water treatment facility, whereas Cd, Pb, and Co had lower concentrations (< 0.1 µg/L). The order of the concentrations among the specified PTEs was like Zn˃Cu˃Ni˃Cr˃Cd˃Pb and Co. The minimum turbidity was 0.34, and the maximum was 1.9 NTU. The range of pH in water samples was 6.51—7.47. The conductivity was 1,203—1,445 ms in water samples. These identified MPs were categorized into fiber and fragments. The color of these identified MPs was blue, red, black, green, and transparent. The minimum and maximum size of the MPs was 196 and 4,018 µm, while the average size was 2,751 ± 1,905 µm. The average concentration of MPs per liter of the water treatment plant was 108.88 ± 55.61. The elements listed are C, O, Na, Mg, Al, Si, K, Ca, and Ti. Fe and Zn were the predominant elements seen using EDX. HQ values of the PTEs were less than one for adults and children. The human health risk associated with all detected PTEs revealed that the HQ values exhibit a satisfactory degree of non-carcinogenic adverse health risk. HI values for adults and children age groups were less than one. In most water treatment samples, the carcinogenic value exceeds the threshold value of 10^−6^. The PTEs and MP concentrations in drinking water should be periodically monitored to minimize consumers' environmental pollution and health risks.

## Introduction

Water is a vital component of terrestrial existence. Maintaining the functionality of the environment and the human body system is critical. Water is an essential component of our food and is present in everything, but the amount may vary. With the increasing public knowledge and concern over the presence of potentially toxic elements (PTEs) that might disrupt the endocrine system, the provision of safe drinking water has become a significant priority for humans (Babaei et al., 2014; Keshtkar et al., 2016; Nadafi et al., 2014; Sobhanardakani, 2019). PTEs in drinking water pose a significant risk due to their poisonous nature and ability to build up in the body over time. This makes them widespread and dangerous for environmental contamination (Chowdhury et al., 2016; Dobaradaran et al., 2018; Hosseini et al., 2020; Sobhanardakani, 2017). Contaminants, such as PTEs, are introduced into drinking water systems due to industrial and natural pollution causes. Insufficient treatment of drinking water would pose a threat to human well-being (Janaszek & Kowalik, 2023; Kowalik et al., 2021). As a result of their accumulation in various body tissues and organs, PTEs, including cadmium (Cd), mercury (Hg), lead (Pb), chromium (Cr), zinc (Zn), and copper (Cu), can all contribute to nephritis, anuria, and other kidney disorders in individuals who consume water tainted with them (Mansour & Sidky, 2002). In addition to PTEs, other substances must be eliminated from water before they can be suitable for drinking. Therefore, choosing a method that can remove a substantial amount of water at once is necessary. PTEs provide various health hazards, thereby necessitating their elimination throughout the water treatment procedure. Elevated quantities of PTEs in drinking water over the threshold set by the United States Environmental Protection Agency (USEPA) may adversely impact human health (EPA, 2018). Water pollution and its impacts are rising, making it a worldwide concern (Bhat & Gaga, 2022; Patidar et al., 2023; Stovall & Bratton, 2022). The hazardous properties of Pb and Cd may result in harmful outcomes such as abortion, early birth, neural damage, low birth weight, renal problems, and hypertension (Järup, 2003; Sarvestani & Aghasi, 2019). Cu buildup in drinking water may lead to Alzheimer's disease (Kaplan et al., 2011).

 Multiple techniques are available for eliminating this particular kind of pollution. Various treatment methods may eliminate PTEs from drinking water, including precipitation using carbonates, sulfides, or organic sulfides; coagulation and flocculation; and membrane treatments, which are efficient in removing both metal anionic and cationic species (Atari et al., 2019). Additional removal methods include electrochemical techniques and selective substitution via ion exchange resins (Johnson et al., 2008). Table 1 provides the threshold values for specific metals and metalloids, as well as pH, conductivity, and turbidity, according to the guidelines set by the World Health Organization (WHO), the USEPA, and the rules established by the Polish government. Specialized laboratories perform analyses on drinking water quality according to the requirements specified in the relevant law. Nevertheless, individuals sometimes voice discontentment over the quality of tap water, often attributed to its taste, appearance, or smell. Moreover, aquatic plants' water composition might differ from tap water's, mainly due to water transport systems. Due to its significance, multiple research has been conducted to examine the long-term health impacts of PTEs from drinking water intake on a global scale (Sarvestani & Aghasi, 2019; Turdi & Yang, 2016).

**Table 1:** Limit values for selected parameters in drinking water

Parameter	WHO (WHO, 2022)	USEPA (EPA, 2018)	Polish regulation (Polish, 2017)
Cd (μg/ L)	3	5	5
Cu (mg/L)	2	1.3	2
Cr (total) (mg/L)	0.05	0.1	0.05
Ni (mg/L)	0.07	0.1	0.02
Pb (mg/L)	0.01	0.015	0.01
Zn (mg/L)	4	5	-
Co (ppb)	-	70	-
Turbidity (NTU)	0.5	< 5	1
pH	6.5 – 8.5	6.5 – 8.5	6.5 – 9.5
Conductivity (μS/cm)	**400**	**2,500**	**2,500**

Nowadays, plastic objects are widely used in several aspects of modern life, such as clothing, cosmetics, healthcare, transportation, communication, and food packaging (Bhat et al., 2021, 2023a; Huppertsberg & Knepper, 2018; Thacharodi et al., 2024b, 2024a). Due to the material characteristics of plastics, which make them seldom degrade, they stay in the environment for a very long period (Bhat et al., 2022, 2023b; Eraslan et al., 2023; Sighicelli et al., 2018) and are a potential hazard to the environment due to their ubiquitous presence (Bhat, 2023a; Bhat et al., 2022; Eraslan et al., 2021; Thacharodi et al., 2024b). This highlights the need to investigate the potential hazards that plastic particles may pose to people and other living species (Bhat, 2023b, 2024a; Bhat et al., 2023c). The plastic particles with a size of 5 mm or less are referred to as microplastics (MPs) and have received significant attention (Arthur et al., 2009; A. J. Verschoor, 2015; Bhat et al., 2023c). Nanoplastics (NPs), a subset of plastic particles, pose a more significant threat to the environment, people, and living beings owing to their tiny size. Bhat et al. (2023c) defined NPs as less than 1 µm or within 300 to 1000 nm range. They are produced due to the deterioration of plastic goods and may also be created during the aging of MPs breakdown. The manufacturing process or even during the use of the object (Bhat, 2024a). Several investigations have conducted extensive studies on MPs in marine ecosystems (Remy et al., 2015; Santana et al., 2016), freshwater bodies (Free et al., 2014; Xiong et al., 2018), and urban watersheds (Birch et al., 2020; Stovall & Bratton, 2022). Recent research shows that individuals ingest a range of 0.1 to 5 g of MPs every week via different exposure means (Senathirajah et al., 2021). It was also estimated that humans inhale 156—240 MPs daily in indoor houses (Bhat, 2024b) while as university inhabitants are exposed to airborne MPs (≥ 2.5 – 336.89 μm) at inhalation rates of 13.88 – 18.51 MPs/m^3^ and 180 – 240 MPs daily (Bhat, 2024c). When the body consumes MPs they are absorbed and distributed through the circulatory system, entering various tissues and possibly causing different detrimental consequences (da Costa et al., 2016; Yee et al., 2021). Most importantly, oxidative stress, cytotoxicity, and translocation to other tissues (Bhat, 2024d; Bhat et al., 2023c; Prata et al., 2020). Human responses to inhaled MPs may lead to chronic inflammation, such as bronchitis, and allergic reactions like asthma or pneumonia (Kacprzak & Tijing, 2022). In South Korea, MPs were traced in raw water (2.2 ± 1.3 MP/L) and treated drinking water (0.02 ± 0.02 MP/L) (Jung et al., 2022). In the Czech Republic, the results of the occurrence of MPs in raw and treated drinking water showed that MPs were found in all water samples, and their average abundance ranged from 1473 ± 34 to 3605 ± 497 MP/L in raw water and from 338 ± 76 to 628 ± 28 MP/L in treated water (Pivokonský et al., 2020). In Germany, the contamination of consumption tap water with MPs was not detected (Weber et al., 2021).

This study aims to evaluate and track the levels of PTEs (Cd, Cu, Cr, nickel (Ni), Pb, Zn, and cobalt (Co)) and overall MPs in a water treatment facility in Kielce, Poland. Evaluating water samples from various sections of the water treatment facilities to detect the presence of PTEs and MPs will demonstrate the effectiveness of the water treatment plants in eliminating metals and MPs. Examining the presence of PTEs and emerging contaminants (MPs) is crucial for tracking their levels in the distribution system, given their significant potential for damage. The PTEs were examined using an Inductively Coupled Plasma Optical Emission Spectrometer (ICP-OES). At the same time, the MPs were characterized using a stereomicroscope and Scanning Electron Microscopy-Energy Dispersive X-ray Analysis (SEM–EDX). The water samples' turbidity, pH, and conductivity were also studied.

## Materials and methods

### Sampling, sample preparation, and quality assurance/quality control

Water samples were collected from a water treatment plant in Kielce, Poland, in October 2022. The samples were collected in (500 ml) glass bottles. Nine samples were collected from different parts of the water treatment plant and were filtered through a Whatman glass microfiber filter (GF/A-1.6µm-47mm). During the analysis, plastic materials were avoided for quality control of MPs in the samples; only glass material was used. All the solutions used in the study were pre-filtered through the same type of filters used in the actual analysis of samples to remove the contaminants if present. Access to the laboratory was limited. Cotton laboratory attire and nitrile gloves were used to reduce the possibility of contamination (Bhat et al., 2024). The surfaces where the tests were carried out and all the materials used during the analysis were regularly cleansed with 5% nitric acid and deionized water to eliminate any possible MPs. Dark glass bottles (500 ml) were utilized for sampling to reduce the influence of photo-degradation. Before sampling, the sampling bottles were cleaned with filtered Milli-Q DI water and used carefully to avoid contamination. Plastic bottles were not used to reduce the chance of MPs being added to the bottles. A layer of aluminum foil was put between the bottles and screw closures to minimize sample contamination. The water sample was filtered through a Whatman glass microfiber filter, and 200 ml was used for quality and quantitative analysis of MPs. In comparison, 100 ml of filtered water was used for ICP-OES analysis.

### Instrumental analysis

The physical characterization of MPs was done using a computer-driven system of automatic image analysis (stereoscopic microscope Nikon SMZ800, Prior stage, NIS-Elements program). A computer set of the microscope with software enabling comprehensive image analysis through automated and manual measurements and counting objects. Stereomicroscope with a zoom head, with a magnification range from 20 × to 126x, guaranteeing spatial vision of the magnified image. A dedicated color CCD digital camera is connected to the microscope. Halogen illuminator with adjustable light intensity and high power. A motorized measuring table allows precise movement control in the XY or XYZ axes. The control is carried out using a control stick or a computer, enabling convenient scanning of the sample surface, setting precision up to 0.1 μm (WawrzeĔczy et al., 2016). Under the stereomicroscope, the plastic particles were counted as the total MPs. Under the stereomicroscope, MPs were counted, and their physical characteristics, like color, type, and size, were also analyzed based on the previous studies (Bhat, 2023a, 2024a, 2024e) using ImageJ software.

The samples' turbidity, pH, and conductivity were also measured using automatic instruments. Turbidity was measured by a Hach turbidity meter (2100P ISO turbidimeter), pH was measured by an Elmetron pH meter (CPC-505), and conductivity was measured by an Elmetron conductivity meter (CC-551).

The concentrations of PTEs (Cd, Cu, Cr, Ni, Pb, Zn, and Co) in the water samples were measured using a Perkin Elmer Optima 8000 ICP-OES manufactured by PerkinElmer, located in Waltham, MA, USA. This device is very effective for analyzing elements, providing exceptional sensitivity, the ability to analyze several elements at the same time, low detection limits, and precise quantitative measurements for a wide variety of elements.

The micro and nanostructure of the MPs collected on the Whatman glass microfiber filter were examined using scanning electron microscopy (SEM). A Quanta FEG 250 microscope obtained from the FEI Company (Hillsboro, OR, USA) equipped with a Large Field Detector (LFD), Backscattered electron detector (BSED), and an energy-dispersive X-ray microanalyzer (EDS) was used. Before being placed under the microscope, the filters could dry properly under natural room conditions without further sputtering to prevent any influence on their composition. The test was performed at vacuum (30 Pa) using an electron beam of 5.00 kV, a working distance of 9.2 – 10.3 mm, and magnification of 4000x – 16,000 × for image acquisition with the LFD and BSED detector for elemental analysis with the EDS detector.

### Health risk assessment

The process of evaluating health risks associated with exposure to a chemical substance involves four stages: identifying potential hazards, assessing the level of exposure, evaluating the relationship between the dose and the response, and characterizing the overall risk. The exposure assessment relies on determining the amount of the substance that is taken in. In this case, the health risk assessment was conducted using data from previous studies on drinking water (Dashtizadeh et al., 2019a, 2019b; Michalski et al., 2020). The Chronic Daily Intake (CDI, mg/kg × day) was determined by using Eq. 1:

1 $${\text{CDI}}={\text{C}}\frac{{\text{CR}}\times {\text{EF}}\times {\text{ED}}}{{\text{BW}}\times {\text{AT}}}$$

where: C is the average concentration of metals at exposure, mg/L; CR is contact rate, L/day; EF is exposure frequency, days per year; ED is exposure duration, years; BW is body weight, kg; AT is period over which exposure is averaged, days. In Table 2, the values of parameters for CDI calculation are reported. Non-carcinogenic health risk assessment is based on the Hazard Quotient (HQ), calculated using Eq. 2.

2 $${\text{HQ}}=\frac{{\text{CDI}}(\mathrm{non carcinogenic})}{{\text{RFD}}}$$

where RFD is the Reference Dose Factor [mg/kg/day], the Hazard Index (HI) is the HQ sum calculated when several metalloids are studied; the critical value for HQ and HI is 1. The individual excess lifetime cancer risk (IELCR) is calculated for carcinogenic substances using Eq. 3.

3 $${\text{IELCR}}={\text{CDI}}\left({\text{carcinogenic}}\right)\times {\text{SF}}$$

where SF is the Slope Factor (mg/kg/day), total IELCR is calculated when several carcinogenic metalloids are studied. The critical value for IELCR and total IELCR is 10^−6^.

**Table 2:** The values of parameters for exposure assessment calculation

Parameter	Value		
	Non-carcinogenic risk assessment		Carcinogenic risk assessment
	Adult	Child	
CR, L/day	2	1	2
EF, days per year	365	365	365
ED, years	30	6	70
BW, kg	70	15	70
AT, days	**ED × 365**	**ED × 365**	**70 × 365**

### Visualizing data

The study included many statistical analyses including calculations of mean, standard deviation, data analysis, human health risk assessment, and graph plotting. These analyses were performed using Excel-2019, Origin-2018, and SPSS-2022 software.

## Results and discussion

### Potentially toxic elements

An investigation was conducted to examine the fate of PTEs throughout the treatment procedure. Results of Cd, Cu, Cr, Ni, Pb, Zn, and Co mean concentrations in samples from water treatment plants steps are presented in Table 3. Cd, Pb, and Co had lower concentrations (< 0.1 µg/L), while the Zn had the highest concentrations at all the points in the water treatment plant compared with other identified PTEs. Cu has the lowest (6.5 µg/L) and highest (83.9 µg/L) concentration at points 2 and 8. Cr has the lowest (0.4 µg/L) and highest (1.3 µg/L) concentration at points 3, 9, and 4. Ni has the lowest (2.8 µg/L) and highest (6.4 µg/L) concentration at points 5 and 2. Zn has the lowest (98 µg/L) and highest (929 µg/L) concentration at points 5 and 7. The concentration of identified PTEs varied between sample points in the water treatment plant. The order of the concentrations among the specified PTEs was like Zn˃Cu˃Ni˃Cr˃Cd˃Pb and Co.

**Table 3:** The concentration of potentially toxic elements in the water treatment samples

Sample points	Concentration (µg/L) of potentially toxic elements analyzed						
	Cd	Cu	Cr	Ni	Pb	Zn	Co
1	< 0.1	7.4	0.7	6.3	< 0.1	631	< 0.1
2	< 0.1	6.5	0.5	6.4	< 0.1	458	< 0.1
3	< 0.1	10.5	0.4	4.2	< 0.1	402	< 0.1
4	< 0.1	9.1	1.3	3.6	< 0.1	145.8	< 0.1
5	< 0.1	24.3	0.9	2.8	< 0.1	98	< 0.1
6	< 0.1	27.2	0.5	4.6	< 0.1	337.5	< 0.1
7	< 0.1	12.8	0.7	3.6	< 0.1	929	< 0.1
8	< 0.1	83.9	0.5	5.2	< 0.1	477.7	< 0.1
9	< 0.1	47.6	0.4	3.4	< 0.1	695	< 0.1
Avg ± SD	**0.1 ± 1.38E-17**	**25 ± 24**	**0.65 ± 0.27**	**4.45 ± 1.2**	**0.1 ± 1.38E-17**	**465.93 ± 247.91**	**0.1 ± 1.38E-17**

The Cd, Cu, Cr, Ni, Pb, and Co concentration values were below the limit values set by WHO (WHO, 2022), USEPA (EPA, 2018), and Polish regulations (Polish, 2017). This indicates that the examined water is suitable for human consumption. For Zn, the concentration levels were mainly higher than those set by WHO (WHO, 2022) due to the dissolution of Zn from pipes. There is currently no recommended limit for Zn concentration in drinking water based on health considerations. Several research have examined the occurrence of PTEs in drinking water, and the findings of this study align with their conclusions. For example, Stegavik (1970) tested the PTEs pollution in Trondheim, Norway's drinking water distribution network. The findings indicated that the levels of Pb, Cd, Cu, and Zn in the drinking water have been less than the standard level, and there is no concern for public health. Dashtizadeh et al. (2019b) evaluate the concentrations of As, Cd, Cr, Ni, Pb, B, Al, Hg, Mn, Zn, Cu, Fe, Se, and Ba in the tap water of Zahedan, Iran. The average amounts of all PTEs in the study were far below the recommended maximum limits set by the USEPA and WHO for drinking water. Another study from Iran, reported by Sarvestani and Aghasi (2019), has been conducted to evaluate the concentrations of Pb, Cd, and Cu in drinking water samples in Kerman, Iran. The findings indicated that the average levels of Pb in tap water were above the acceptable threshold established by the WHO and the USEPA. The concentrations of As, Cd and Cr in 26 water samples collected from 13 rural areas near mines in Sabzevar, Iran, showed the potential health risks among local residents. As and Cr concentration in drinking water of the rural regions in the vicinity of mines were estimated to be in the range of 0.0152 to 0.0220 and 0.0194 to 0.1806 mg/L, respectively, which exceeded the drinking water guidelines recommended by WHO (Shams et al., 2020).

### Turbidity, pH, and conductivity

The turbidity, pH, and conductivity of the water treatment samples are given in Table 4. Water samples' turbidity, pH, and conductivity were cross-checked with the USEPA, WHO, and Polish drinking water guidelines. In most cases, the parameters followed these standards. The minimum turbidity was 0.34, and the maximum was 1.9 NTU. Turbidity of drinking water should not exceed 5 NTU (EPA, 2018) and should be 1 NTU (Polish, 2017). All drinking facilities should able to achieve 0.5 NTU before disinfection at all times and average 0.2 NTU or less) (WHO, 2022). Turbidity may arise due to substandard quality of the water supply, inadequate treatment, and disruption of sediments and biofilms inside distribution systems, or the infiltration of contaminated water via main breaks and other faults. High turbidity levels may cause the discoloration of materials, fittings, and textiles that are exposed during washing. Additionally, it can disrupt the efficiency of treatment operations. Visible turbidity diminishes the suitability of drinking water. While the majority of particles causing turbidity do not pose a health risk (although they may suggest the presence of harmful chemical and microbiological pollutants), many consumers see turbidity as a sign of danger and deem turbid water unfit for consumption.

**Table 4:** Turbidity, pH, and conductivity of water treatment samples

Sample points	Turbidity (NTU)	pH	Conductivity (ms)
1	1.52	6.8	1,345
2	1.63	7.41	1,350
3	0.6	7.47	1,401
4	0.34	6.98	1,344
5	1.46	7.26	1,425
6	1.9	7.12	1,445
7	1.04	6.51	1,203
8	0.58	6.62	1,204
9	0.9	6.83	1,204
Avg ± SD	1.1 ± 0.54	7 ± 0.34	1324 ± 97

The range of pH in water samples was 6.51—7.47. pH of the drinking water should be 6.5—8.5 (EPA, 2018; WHO, 2022). The water pH in this study followed the pH of 6.6—7.8 found by Dashtizadeh et al. (2019b) in drinking tap water in Zahedan City, Iran. For effective disinfection with chlorine, the pH should be less than 8; however, lower-pH water (approximately pH seven or less) is more likely to be corrosive. It is necessary to regulate the pH of the water that enters the distribution system in order to reduce the corrosion of water mains and pipes in domestic water systems. Alkalinity and calcium management are factors that influence the stability of water and its tendency to corrode pipelines and appliances. Inadequate efforts to reduce corrosion may lead to the pollution of potable water and negative impacts on its flavor and visual qualities.

The conductivity was 1,203—1,445 ms in water samples. The conductivity of the drinking water should be 2,500 ms (Polish, 2017). High or low conductivity levels can indicate potential issues, such as contamination, salinity, or the need for water treatment. The conductivity of water samples in this study was higher than the conductivity (157—794 ms) found by Michalski et al. (2020) in tap water consumption in Silesia, Poland.

## Microplastics

**Fig. 1:**
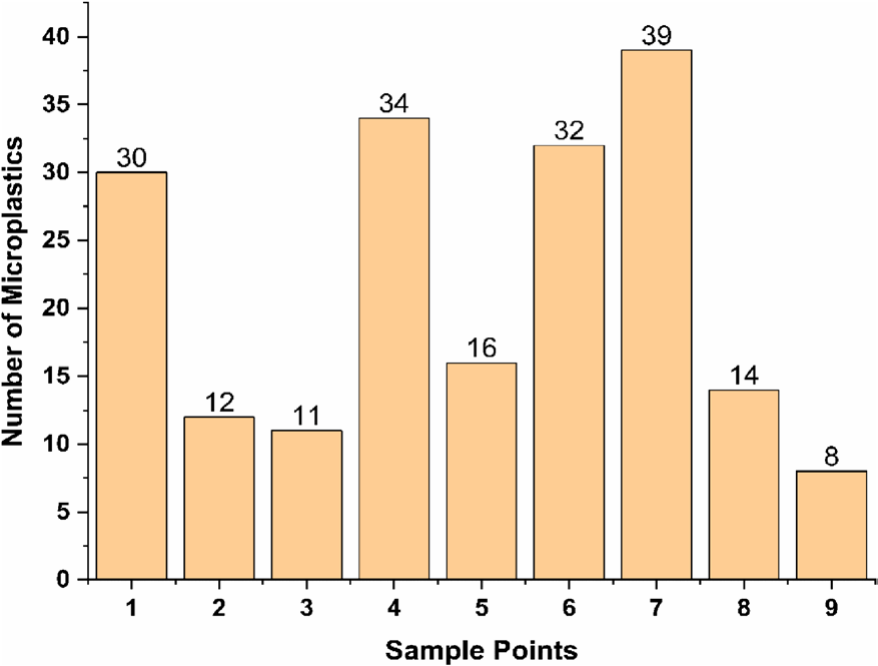
The concentration of microplastics in the water treatment plant

**Fig. 2:**
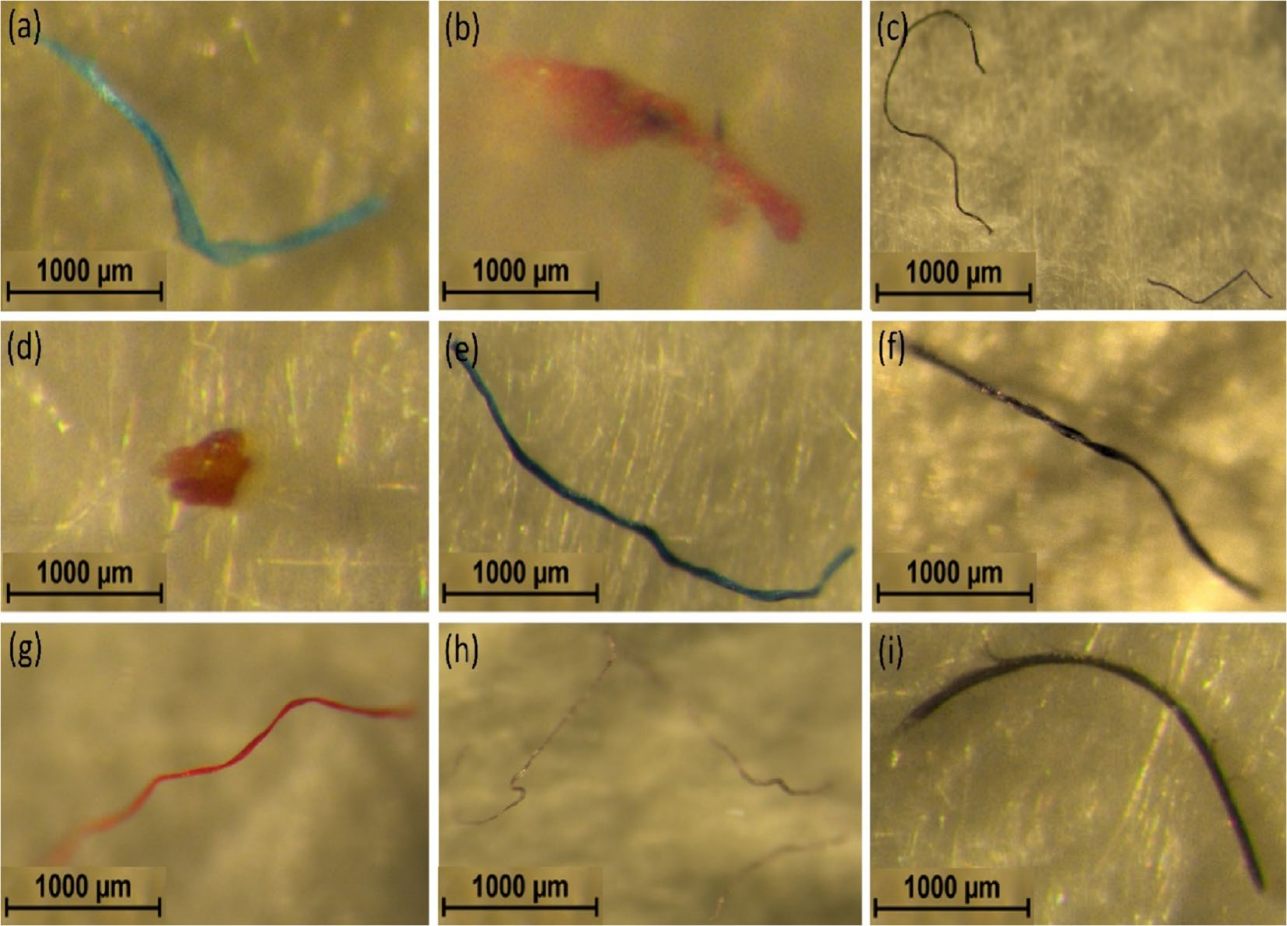
Typical examples of some microplastics with their type and color under the stereomicroscope found in this study. Fiber (a; blue, b; red, c; black,) and (e; green, f; black, g; red, h; transparent, i; black) and fragment (d; red)

The MPs found in the water treatment plant are shown in Fig. 1. The concentration of MPs varies among the sample points. Sample point 7 had the highest MPs (39 ± 195), and sample point 9 had the lowest (8 ± 40). The average MPs were 21 at each point in the water treatment plant, and 196 ± 980 in 100 ml MPs were found there. The average concentration of MPs per liter of the water treatment plant was 108.88 ± 55.61. MPs were categorized into size, shape, and color (Fig. 2). These identified MPs were categorized into fiber and fragments. The color of these identified MPs was blue, red, black, green, and transparent. The minimum and maximum size of the MPs was 196 and 4,018 µm, while the average size was 2,751 ± 1,905 µm.

MPs have been in different water bodies, but limited studies have been done on water treatment plants. The MP concentration (108.88 ± 55.61) found in this study was higher than the number of MPs found in raw water and drinking water treatment plants 0.5 to 7 and 0.02—0.11 particles/L in Busan, South Korea (Jung et al., 2022). However, in other studies, 1,000—6,000 particles/L and several hundred particles/L were identified in raw water (upstream) and treated drinking water, respectively (Kankanige & Babel, 2021; Pivokonský et al., 2020; Wang et al., 2020). Drinking water treatment can effectively block various water-borne particles, potentially including MPs. However, plastic components in treatment plants and distribution networks may erode or degrade, leading to MPs in drinking water. Plastic bottles and caps used for bottled water may contain MPs that may end up in drinking water (Oßmann et al., 2018). MP's size, shape, and polymer type may affect their transportation in water and may impact the toxicity and effectiveness of drinking-water treatment. (WHO, 2019).

### SEM–EDX

**Fig. 3:**
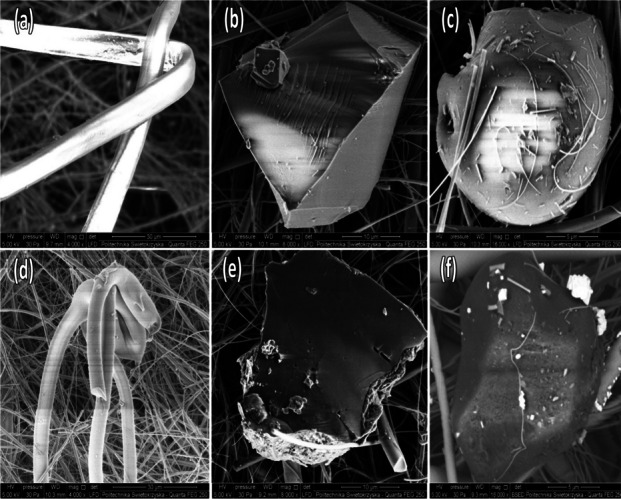
Scanning electron microscope images of microplastics (sourced from nine raw water sources) deposited on the Whatman glass microfiber filter

SEM examples of different-shaped MPs (fiber, pellet, and fragment) are shown in Fig. 3. The EDX analysis of MPs is shown in Table 5. The elements listed are C, O, Na, Mg, Al, Si, K, Ca, and Ti. Fe and Zn were the predominant elements seen using EDX. The EDX study revealed that C, O, and Si exhibited the highest concentrations, while the other detected elements had the lowest concentrations. The distribution and composition of elements in MPs varied across the samples, with no consistent presence or dominance of any particular element or proportion. The variance might arise from identifying distinct MPs and their compositions or from pollutants on their surface (Bhat, 2023a; Bhat et al., 2023b). C, O, and plastic-specific chemical components in high concentrations prove MPs' identity. The primary components of silicate minerals (such as clays) found on MPs include elements Al, Si, Na, and Mg. These elements are likely present due to the adsorption of silicates onto the surface of the MPs. The elements Al, Ca, Si, and Mg mostly derive from natural sources such as soil or dust, whereas Cu and Zn come from human activities such as burning fossil fuels and vehicle abrasion (Abbasi et al., 2020; Arslan, 2001) can also adhere to the surface of MPs. The elements Na, Mg, K, Al, Si, Ca, Cl, and O stick to the surface of the MPs, and silicate minerals such as clays may cause their presence (Ganesan et al., 2019; Nematollahi et al., 2022). Zn is a well-recognized urban element believed to have been generated mostly from human activities, such as transportation and industrial operations (Ahmady-Birgani et al., 2015; Nematollahi et al., 2021). Fe is often used as an addition in plastic materials to attain certain qualities, such as imparting color to the plastic (Nematollahi et al., 2020). A broad range of elements (Ti, Si, Zn, Al, and Fe) have been employed in paintings as pigments, binders, or additions to generate a wide range of colors, textures, and functionalities (Kowalczyk et al., 2012; Lopez et al., 2023; Pfaff, 2021; Zuin et al., 2014). These additives may be released into the environment due to weathering since they are not chemically bound to the polymeric matrix (Bhat, 2024d; Bhat et al., 2023b). Minerals like gypsum contain S naturally (Kong et al., 2020), and these gypsums are used inside houses.

**Table 5:** EDX analysis of microplastics showing elemental composition

MP 1		MP 2	MP 3	MP 4	MP 5	MP 6	MP 7	MP 8	MP 9	MP 10	MP 11	MP 12	MP 13	MP 14	MP 15	MP 16
Element	Wt %	Wt %	Wt %	Wt %	Wt %	Wt %	Wt %	Wt %	Wt %	Wt %	Wt %	Wt %	Wt %	Wt %	Wt %	Wt %
C	57.3	42.8	28.6	39.2	45	50.9	41	0	1.63	1.61	1.34	1.26	30.4	15.7	34	26.4
O	33.8	36.3	36.6	36.2	33.3	27	27.3	49.5	41.3	43.2	43	40.2	38.2	40.9	40.4	38.3
Na	1.93	4.57	5.75	5.02	4.45	3.29	4.2	4.53	11.1	10.1	10.2	8.84	5.66	7.41	3.41	5.48
Mg	0	0.19	0	0.17	0.3	0.29	0.22	0.87	2.55	0	0.46	0.31	0.57	0.35	0.53	0.35
Al	0.67	1.46	2.73	1.83	1.62	1.33	1.95	1.17	1.71	4.12	4.28	4.17	2.26	3.36	0.89	1.67
Si	4.59	11.2	17.2	12.5	10.4	9.2	15.7	42.2	36.7	32.1	32	34.9	17	24.4	6.31	13.6
P	0.03	0	0	0	0	0	0	0	0	0	0	0	0	0	0.23	0.18
S	0	0	0.46	0.23	0.23	0.18	0.2	0	0	0	0	0	0	0	0.39	0.31
Cl	0	0.14	1.67	0.71	0.31	0.33	0.36	0	0	0	0	0	0	0.34	0.41	0.36
K	0.35	0.92	2.41	1.41	1.21	0.94	1.71	0.62	0.71	2.93	2.72	3.38	1.63	2.38	1.4	2.06
Ca	0.2	0.43	1.25	0.66	0.91	4.55	3.67	1.1	4.17	1.32	1.33	1.52	0.86	1.27	10.73	8.73
Ti	0.27	0.65	0.89	0.53	0.49	0.4	0.81	0	0	1.21	1.25	1.44	0.78	1.12	0.24	0.48
Fe	0.21	0.27	0.38	0.22	0.19	0.35	0.39	0	0	0.29	0.42	0.42	0.29	0	0.25	0.3
Cu	0	0	0	0	0	0	0.46	0	0	0	0	0	0	0	0	0.3
Zn	0.61	0.93	1.93	1.21	1.15	1.12	1.85	0	0	3.02	2.86	3.49	1.7	2.59	0.72	1.3
Total	100	100	100	100	100	100	100	100	100	100	100	100	100	100	100	100

### Health risk assessment

The determination of PTE's non-carcinogenic and carcinogenic effects was done using the HQ and ELCR. Table 6 summarizes the estimated HQ of trace elements for two age groups (children and adults) that consume the drinking water in the study area. Table 6 shows that all HQ values of the PTEs were less than one for adults and children. Consequently, evaluating the human health risk associated with all detected trace elements revealed that the HQ values exhibit a satisfactory degree of non-carcinogenic adverse health risk (Ghaderpoori et al., 2018; Turdi & Yang, 2016). Table 5 also shows that HI values for adults and children age groups were less than one and were in the order of Zn 0.035788606 > Cu 0.000262053 > Ni 2.29139E-05 > Cr 5.05707E-07 and Zn 0.083508418 > Cu 0.00061147 > Ni 5.34669E-05 > 1.18001E-06 respectively. Based on the non-carcinogenic risk, these HI values of less than one show that the drinking water of the water treatment plant is safe for human ingestion. Overall, the study's findings showed no appreciable non-carcinogenic risk for the PTEs examined; nevertheless, regular monitoring is still required since unexpected contamination may occur. Table 6 represents the estimated ELCR values of carcinogenic PTEs. For carcinogenic substances, the acceptable threshold is 10^−6^. In most water treatment samples, the carcinogenic value exceeds the threshold value. The total HI values were above one in the drinking water of all villages in Joghatai, Iran, and generally, total HI values for children were much higher than those of adults in each studied area (Shams et al., 2020), and carcinogenic risk for Cr in drinking water of all villages for children was above the USEPA risk assessment guideline limit. Sajjadi et al. (2022) found children were more exposed to health risks due to drinking water containing As, Cr, and Pb in Ghayen County, Iran.

**Table 6:** Non-carcinogenic and carcinogenic risk of potentially toxic elements in water treatment plant

**Sample points**	HQ/ Adult				HQ/ Child				IELCR
	HQ/Cu	HQ/Cr	HQ/Ni	HQ/Zn	HQ/Cu	HQ/Cr	HQ/Ni	HQ/Zn	Cr
1	8.45702E-06	5.99991E-08	3.59995E-06	0.005411062	1.97334E-05	1.40001E-07	8.40004E-06	0.012626063	1.6E-05
2	7.42846E-06	4.28565E-08	3.65709E-06	0.003929084	1.73334E-05	1.00001E-07	8.53338E-06	0.009168046	1.14286E-05
3	1.19998E-05	3.42852E-08	2.39996E-06	0.003447377	2.80001E-05	8.00004E-08	5.60003E-06	0.00804404	9.14286E-06
4	1.03998E-05	1.11427E-07	2.05711E-06	0.001249696	2.42668E-05	2.60001E-07	4.80002E-06	0.002916015	2.97143E-05
5	2.7771E-05	7.71417E-08	1.59998E-06	0.000839987	6.48003E-05	1.80001E-07	3.73335E-06	0.00196001	2.05714E-05
6	3.10852E-05	4.28565E-08	2.62853E-06	0.002892814	7.25337E-05	1.00001E-07	6.13336E-06	0.006750034	1.14286E-05
7	1.46284E-05	5.99991E-08	2.05711E-06	0.007964452	3.41335E-05	1.40001E-07	4.80002E-06	0.018584093	1.6E-05
8	9.58843E-05	4.28565E-08	2.97138E-06	0.00409451	0.000223734	1.00001E-07	6.93337E-06	0.009554048	1.14286E-05
9	5.43992E-05	3.42852E-08	1.94283E-06	0.005959625	0.000126934	8.00004E-08	4.53336E-06	0.01390607	9.14286E-06
HI = ΣHQ	0.000262053	5.05707E-07	2.29139E-05	0.035788606	0.00061147	1.18001E-06	5.34669E-05	0.083508418	Total ELCR 0.000134857

## Conclusion

The presence of PTEs in drinking water poses a significant concern due to their potential adverse health effects on consumers. This study presents a comprehensive assessment of PTEs such as (Cd, Cu, Cr, Ni, Pb, Zn, and Co) and MPs in drinking water, considering a wide range of parameters, including turbidity, pH, conductivity, and health risk assessment. Zn had the greatest quantities among all the PTEs in the water treatment plant, whereas Cd, Pb, and Co had lower values, measuring less than 0.1 µg/L. The concentrations of the required PTEs followed the order Zn > Cu > Ni > Cr > Cd, Pb, and Co. The lowest turbidity recorded was 0.34 NTU, while the highest was 1.9 NTU. The pH values observed in the water samples ranged from 6.51 to 7.47. The conductivity of the water samples ranged from 1,203 to 1,445 ms. MPs are currently regarded as pervasive contaminants due to their extensive presence in all environmental components, particularly water sources. The increasing amount of plastic waste has raised concerns about these MPs in aquatic environments. MPs can be fragmented into NPs that can pass through water treatment processes and into tap water, potentially threatening human health because of their high adsorption capacity for hazardous organic materials and their intrinsic toxicity. The detected MPs were classified into two groups: fiber and fragments. The detected MPs were blue, red, black, green, and transparent. The MPs exhibited a range of sizes, with the smallest being 196 µm and the largest being 4,018 µm. The average size of the MPs was 2,751 µm, with a standard deviation of 1,905 µm. The mean MP concentration per liter of the water treatment facility was 108.88 ± 55.61. The listed elements are C, O, Na, Mg, Al, Si, K, Ca, and Ti. The elements Fe and Zn were the most prevalent when seen using EDX. The concentrations of PTEs in both adults and children were below one. The assessment of human health risks related to PTEs indicates that the HQ values demonstrate an acceptable level of non-carcinogenic adverse health risk. The HI values for adults and children in all age categories were below one. The carcinogenic value in most water treatment samples is above the threshold value of 10^−6^. Therefore, it is advisable to regularly test the levels of metals and metalloids, pH, turbidity, conductivity, and MPs outside the scope of statutory regulations to limit potential health hazards for consumers. Although the study analyzed several potentially toxic elements, it did not cover all possible contaminants that could be present in drinking water, potentially overlooking other significant pollutants. Explore and develop effective mitigation strategies at water treatment plants to reduce the levels of PTEs and MPs, ensuring safe drinking water for consumers.

## Data Availability

Data will be made available on request.
